# The diagnostic value of SPECT/CT in predicting the occurrence of osteonecrosis following femoral neck fracture: a prospective cohort study

**DOI:** 10.1186/s12891-020-03538-1

**Published:** 2020-08-03

**Authors:** Jae Youn Yoon, Soong Joon Lee, Kang Sup Yoon, Pil Whan Yoon

**Affiliations:** 1 Department of Orthopedic Surgery, Dongguk University Ilsan Hospital, Gyeonggi-do, Republic of Korea; 2 Department of Orthopedic Surgery, SMG-SNU Boramae Medical Center, Seoul National University College of Medicine, Seoul, Republic of Korea; 3 Department of Orthopedic Surgery, University of Ulsan College of Medicine, Asan Medical Center, 88 Olympic-ro 43-gil, Songpa-gu, Seoul, 05505 Republic of Korea

**Keywords:** Osteonecrosis, Arthroplasty, replacement, hip, Femur neck fracture, Tomography, emission-computed, single-photon, Tc-99 m-HDP

## Abstract

**Backgrounds:**

One of the most significant complications after a femoral neck fracture is osteonecrosis of the femoral head (ONFH). The concomitant use of single-photon emission computed tomography (SPECT) with computed tomography (CT) increases the sensitivity for detecting the anatomic location and severity of ONFH. In this study, we evaluated the diagnostic value of SPECT/CT for the occurrence of ONFH by quantifying the perfusion status of the femoral head.

**Methods:**

A total of 30 patients who had multiple pinnings for femur neck fractures were included in this study. We classified the perfusion status into three groups: normal perfusion, decreased perfusion, and avascular groups, and compared the occurrence of femoral head necrosis between them. For quantitative analysis, we evaluated the uptake ratio of both femur heads (head-to-head uptake ratio). If the patient’s contralateral hip was incomparable, we measured the uptake ratio from the superior dome of the ipsilateral acetabulum (head-to-acetabulum uptake ratio).

**Results:**

Twenty-four patients out of 30 achieved bone union, whereas the others developed ONFH. When the population was divided into intact and defective perfusion groups on scintigraphy, the sensitivity, specificity, and accuracy of the test were 83.3, 75.0, and 76.7%, respectively. The mean head-to-head uptake ratio value with a 95% confidence interval (CI) was 1.10 (95% CI: 0.85–1.36). In the osteonecrosis group, the mean value of the head-to-head uptake ratio was 0.33 (95% CI: 0.28–0.38). In contrast, the ratio was 1.30 (95% CI: 1.03–1.57) in the non-osteonecrosis group, demonstrating a significant difference in the uptake ratio (*P <* 0.001). When the cutoff value of the uptake ratio was set to 0.5, both the sensitivity and specificity were 100%. There was also a significant difference in the head-to-acetabulum uptake ratio between the two groups (*P* <  0.001).

**Conclusions:**

SPECT/CT was useful in evaluating the perfusion status of the femoral head, showing high accuracy in predicting the occurrence of avascular necrosis. To demonstrate the reliability and validity of SPECT/CT, further prospective studies on a larger scale are warranted.

## Background

The incidence of femoral neck fractures (FNFs) is increasing as the number of elderly people increases. Despite developments in treatment options, FNF still has a high complication rate, which leads to an economic burden to society and necessitates guidelines for successful outcomes [1–3]. The surgical treatment of FNF includes osteosynthesis or arthroplasty, which can be selected based on the displacement of fracture or the age of the patient [4–9]. Several studies reported the incidence of osteonecrosis of the femur head (ONFH) after the osteosynthesis of FNF to be 12.0–17.3% [7, 10]. Other studies also reported that ONFH occurs in about 9.9–33% of the displaced fractures and 6.4–10.8% of the nondisplaced fractures [11–14].

Perfusion failure is considered the primary mechanism responsible for ONFH. Therefore, the exact evaluation of the perfusion status of the femoral head after FNF is critical in predicting the risk of ONFH so that it could guide a surgeon to select the optimal treatment modalities to lower the complication rate and achieve a favorable outcome. To date, various diagnostic methods, including laser Doppler flowmetry, ultrasonography, arterial angiography, intra-osseous venography, intra-osseous oxygen pressure measurement, and scintigraphy, have been introduced to assess perfusion status. Among them, scintigraphy is generally considered the modality of choice [15–18]. Scintigraphy has an 87–95% accuracy in predicting the occurrence of ONFH or nonunion. However, most of the study results are based on qualitative measurements. Conventional bone scans provide poor image descriptions and have limitations in precisely locating the lesion, which lowers the specificity of the test [16]. Furthermore, the two-dimensionally reconstructed three-dimensional anatomical structure is likely to produce false-negative results due to overlying structures.

Single-photon emission computed tomography (SPECT) with computed tomography (CT) using a hybrid camera system enables the clinicians to find the exact location of the lesion and assess the severity as well. Several studies also examined the diagnostic accuracy of SPECT/CT [17, 18]. Despite the virtues, SPECT/CT still depends on the subjective judgment of the specialists and non-quantitative methods for assessing the image, which diminishes the objectivity and accuracy of the test [19].

To our knowledge, only one study has reported a quantitative analysis of SPECT/CT to predict ONFH in patients with FNF treated with internal fixation [20]. However, their method is only available to patients with FNF with a healthy contralateral hip because they used the photon number ratio in the affected side against the unaffected side. Therefore, we evaluated the diagnostic value of SPECT/CT in assessing the risk of ONFH in patients with FNF. We used three different methods to determine femoral head vascularity: 1) qualitative analysis by specialists board-certified in nuclear medicine, 2) quantitative analysis using the photon uptake ratio in the affected femoral head against the unaffected femoral head, and 3) quantitative analysis using the photon uptake ratio in the affected femoral head against the superior acetabular dome of the affected hip.

## Methods

### Subjects

The study was a prospective cohort study performed at a single center with 145 patients with FNFs from January 2010 to March 2012 under the approval of the Institutional Review Board. All patients were fully informed of the concept of the study, generally accepted treatment methods and the prognosis of both multiple pinning and arthroplasty before participation in the study. Among the initially enrolled 145 patients, we excluded 90 patients from the study who chose to undergo total hip arthroplasty (THA) or bipolar hemiarthroplasty (BPHA) instead. Of the 52 patients, we also excluded 22 patients due to a lack of preoperative SPECT/CT evaluations and a short follow-up length of less than two years. Finally, 30 patients were enrolled in this study, including 11 men and 19 women with a mean age of 64.3 years (range, 13 to 90 years). The average duration of follow-up was 4.0 years (range, 2.0 to 8.9 years). We used Garden’s classification to classify the type of FNFs. There were 17 (56.7%) Garden I patients; four (13.3%) Garden II patients; six (20%) Garden III patients; and three (10%) Garden IV patients [21].

### Surgical technique and postoperative management

We performed all operations within 48 h following the initial trauma and surgically treated the patients with closed-reduction and internal fixation using three cannulated screws on a fracture table. The patients maintained partial weight-bearing using crutches or walker assistance for six weeks postoperatively. Full weight-bearing was allowed gradually after six weeks. All patients were followed-up in the clinic with serial radiographs at regular intervals (postoperative six weeks; three, six, twelve months, and annually thereafter). Since the screws used to treat fractures are made of stainless steel, we had a limitation in the detection of ONFH using magnetic resonance imaging (MRI). We, therefore, traced the occurrence of ONFH using simple radiographs (total hip anteroposterior & translateral view) of the patients. All radiographs were analyzed by a board-certified radiology specialist who was blinded to the SPECT/CT results to detect the occurrence of ONFH. The radiographical diagnostic criteria for ONFH are 1) patch sclerosis and/or subchondral cyst formation, 2) crescent sign and/or irregular bone density at femoral head, 3) collapse or flattening of femoral head. We also confirmed the diagnosis of ONFH histopathologically by undergoing bone biopsy of the surgical specimens of patients who underwent total hip arthroplasty (THA) due to ONFH complications.

### SPECT/CT evaluation and analysis

Preoperative SPECT/CTs were obtained within 48 h of admission to the hospital using a dual-headed gamma camera (Infina, Hawkeye 4, GE Healthcare, Milwaukee, WI, USA) equipped with low-energy high-resolution (LEHR) collimators. The SPECT data were acquired with 60 stops over 180 at 12 s per stop using a 128 × 128 matrix within three to five hours post-injection of 1100 MBq Tc-99 m HDP. The CT acquisition was performed at 140 kvp and 2.5 mA using a 512 × 512 matrix and a 5-mm slice thickness (pitch 10, interval 2.95 mm). The SPECT images were reconstructed using an iterative algorithm provided by the manufacturer to reduce starring artifacts by bladder activity. And attenuation correction was done with CT-derived attenuation maps.

#### Qualitative analysis

**Fig. 1:**
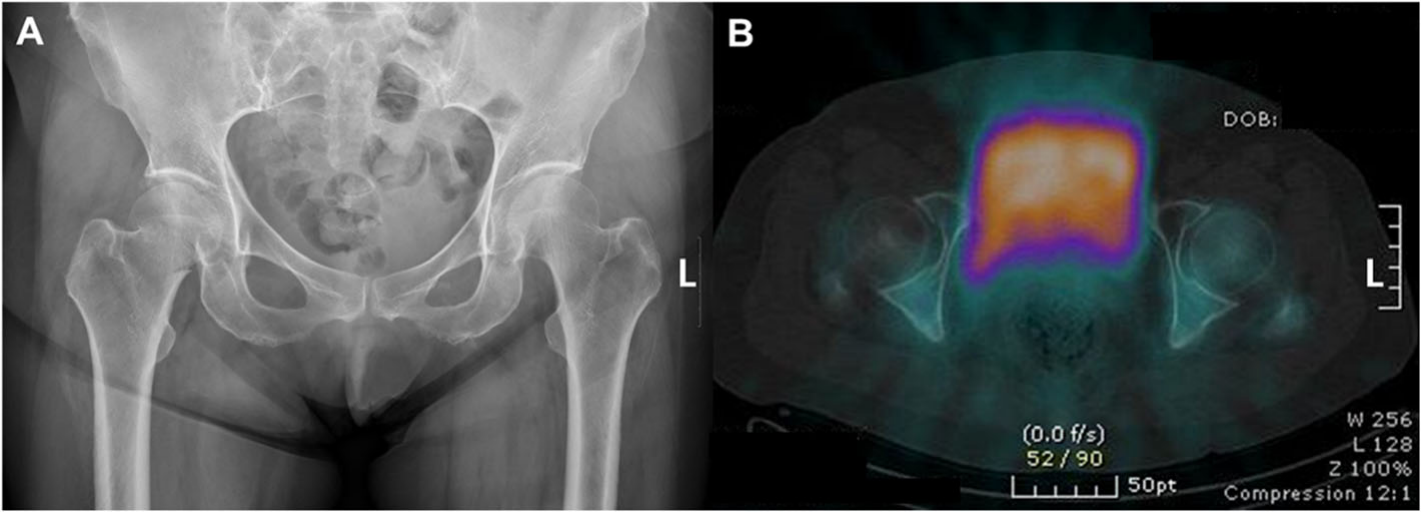
Qualitative analysis of SPECT/CT. **a** Right femur neck fracture is diagnosed by simple radiograph. **b** Photon uptake of the right femur head is decreased compared to the left femur head

For the qualitative analysis, two experienced board-certified nuclear medicine specialists, who were blinded from the patients’ clinical information, classified the perfusion status of the femoral head on the SPECT/CT scans into two groups: the normal perfusion group and the avascular group. Inter-observer discrepancies were resolved through discussion (Fig. 1).

#### Quantitative analysis: head-to-head uptake ratio

**Fig. 2:**
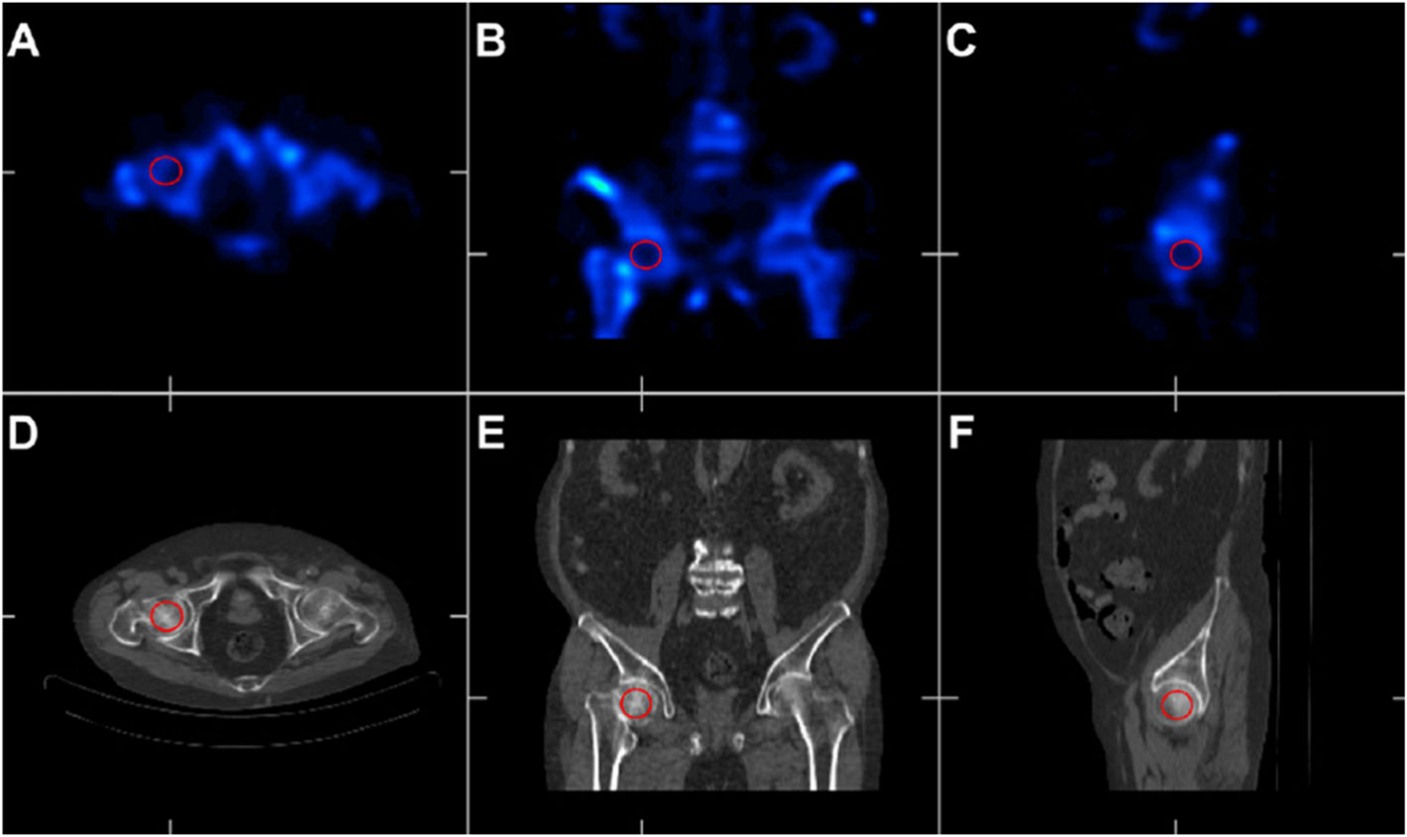
Quantitative analysis of the head-to-head uptake ratio with ROI in a 3-cm circle, excluding the cortical bone of femur head. **a**,**d** ROI measured in axial view of SPECT/CT. **b**,**e** ROI in coronal view. **c**,**f** ROI in sagittal view. Three independently measured values of pixels are summated to an average value of ROI

We used the workstation for quantitative analysis. Scintigraphy images are subject to the amount of injected radionuclide and the variability in its distribution depending on the time interval between the injection and the time point of the test and renal and hepatic function, and differences can be seen even between individuals with normal perfusion [22–24]. Therefore, we tried to compensate for the variability of the measured value by choosing a relative method for the quantitative analysis. We first defined a 3-cm-sized three-dimensional (3-D) circle as the region of interest (ROI), of which the center was aimed at the center of the femoral head, containing maximum cancellous bone without including the cortical bone. Next, we determined the mean photon uptake of the affected femoral head by averaging the photon uptake numbers in the ROI on the coronal, axial, and sagittal planes. The mean photon uptake of the unaffected femoral head was also measured by the same method. Then, the head-to-head uptake ratio was calculated by dividing the value of the affected hip by that of the unaffected hip (Fig. 2).

#### Quantitative analysis: head-to-acetabulum uptake ratio

**Fig. 3:**
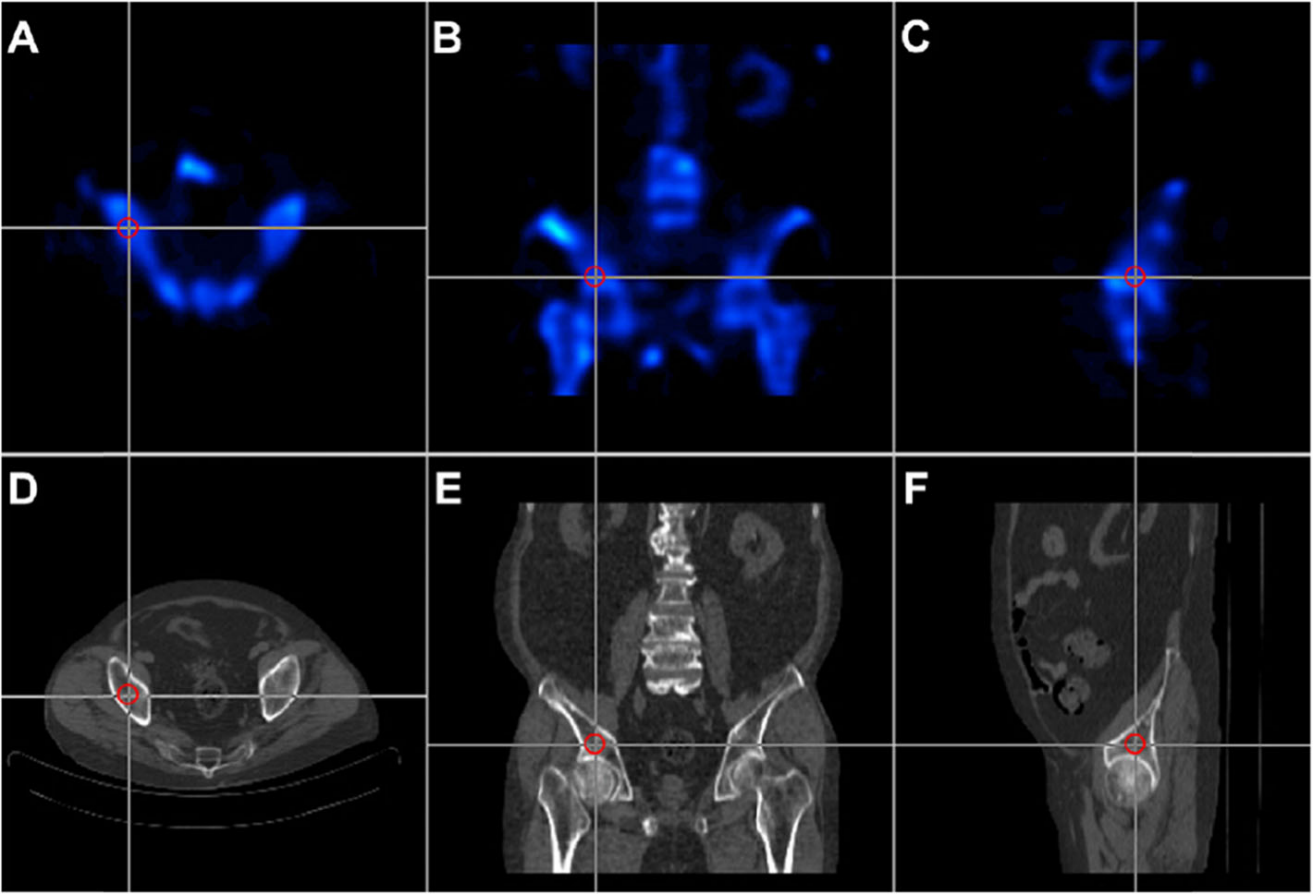
Quantitative analysis of the head-to-acetabulum uptake ratio with ROI in a 1-cm circle at the dome of the acetabulum in the affected hip. **a**,**d** ROI measured in axial view of SPECT/CT. **b**,**e** ROI in coronal view. **c**,**f** ROI in sagittal view. Three independently measured values of pixels are summated to an average value of ROI

When the presence of metal implants or ONFH at a contralateral hip was confirmed, we substituted the analysis from the head-to-head ratio to the head-to-acetabulum ratio by evaluating the photon uptake numbers from the contralateral hip to the ipsilateral acetabular dome. Unlike the femoral head, the acetabular dome is aspherical and surrounded by thicker pelvic cortices, which allows relatively smaller space for applying the ROI. The pre-measured maximum ROI diameter from computed tomography of each patient varied widely from 1.0 to 2.4 cm, depending on their pelvic size and morphology. Therefore, to set the applicable value for all enrolled patients, the ROI at the acetabular dome was defined as a 1-cm-sized 3-D circle containing cancellous bone as much as possible without involving the cortex. The mean photon uptake of the acetabular dome was determined by averaging the photon uptake numbers on the coronal, axial, and sagittal planes (Fig. 3).

### Statistical analysis

Based on the final follow-up, we divided the patients into ONFH and non-ONFH groups. The sensitivity and specificity of the qualitative evaluation of the occurrence of osteonecrosis were assessed by crossover analysis. The Mann-Whitney test was used for analyzing numerical data and Fisher’s exact test was used for categorical data. A receiver operating characteristics (ROC) curve was plotted from the quantitative analysis data of two groups in order to derive a cut off value for predicting ONFH. Area under curve (AUC) of Two ROC curves were compared using Delong’s test for statistical significant change. Statistical analyses were proceeded using SPSS statistical software version 21 (IBM Co., Armonk, NY, USA), and a *P*-value of < 0.05 was considered statistically significant.

## Results

At the final follow-up, osteonecrosis developed in six patients out of 30 (16.7%), whereas bone union was confirmed in the other 24 patients without complications. The mean interval of time from surgery to the diagnosis of ONFH was 8.1 months (range, 4.6 to 17 months). The mean age of the ONFH group was significantly younger than that of the non-ONFH group, and the ONFH group also had a higher rate of fracture displacement (Table 1). Other demographic factors showed no significant differences between the groups.

**Table 1:** Patient demographics between the two groups

Variables (mean, 95% CI)	Total (*N* = 30)	ONFH (N = 6)	Non-ONFH (*N* = 24)	*p*-value
**Age (years)**	64.3 (57.3–71.3)	44.2 (28.3–60.1)	69.3 (62.7–75.9)	0.009
**Female (%)**	63.3% (*n* = 19)	66.7% (*n* = 4)	62.5% (*n* = 15)	0.900
**BMI (kg/m2)**	21.8 (20.6–23.0)	22.8 (20.4–25.2)	21.5 (20.1–23.0)	0.273
**BMD (g/cm2)**	0.7 (0.7–0.8)	0.9 (0.7–1.0)	0.7 (0.6–0.8)	0.093
**Displaced fracture (%)**	30% (*n* = 9)	100% (*n* = 6)	12% (*n* = 3)	< 0.001
**Duration of follow-up (years)**	2.4 (2.2–2.5)	2.1 (2.0–2.2)	2.4 (2.3–2.6)	0.082

*BMI* body mass index; *BMD* bone marrow density, *CI* confidence interval, *ONFH* osteonecrosis of the femur head

### Qualitative evaluation of SPECT/CT

Eleven patients showed a loss of perfusion, whereas the remaining 19 patients showed normal perfusion. Six out of the 11 patients who showed a loss of perfusion, achieved bone union, whereas the other five patients were diagnosed with ONFH at the final follow-up. Eighteen out of the 19 patients classified into the normal perfusion group achieved bone union, and only one patient was diagnosed with ONFH. The sensitivity, specificity, and diagnostic accuracy of the prediction for the occurrence of osteonecrosis were 83.3, 75.0, and 76.7%, respectively. The positive likelihood ratio and negative likelihood ratio were 3.33 (95% confidence interval; CI: 1.52–7.27) and 0.22 (95% CI: 0.04–1.35) (Table 2).

**Table 2:** Sensitivity and specificity for the prediction of the occurrence of ONFH

Qualitative evaluation	ONFH (***N*** = 6)	Non-ONFH (***N*** = 24)	Subtotal
**Decreased or no photon uptake**	5	6	11
**Normal photon uptake**	1	18	19
**Subtotal**	6	24	30

*ONFH* osteonecrosis of the femur head

### Quantitative evaluation of SPECT/CT

#### Evaluation of head-to-head uptake ratio

**Fig. 4:**
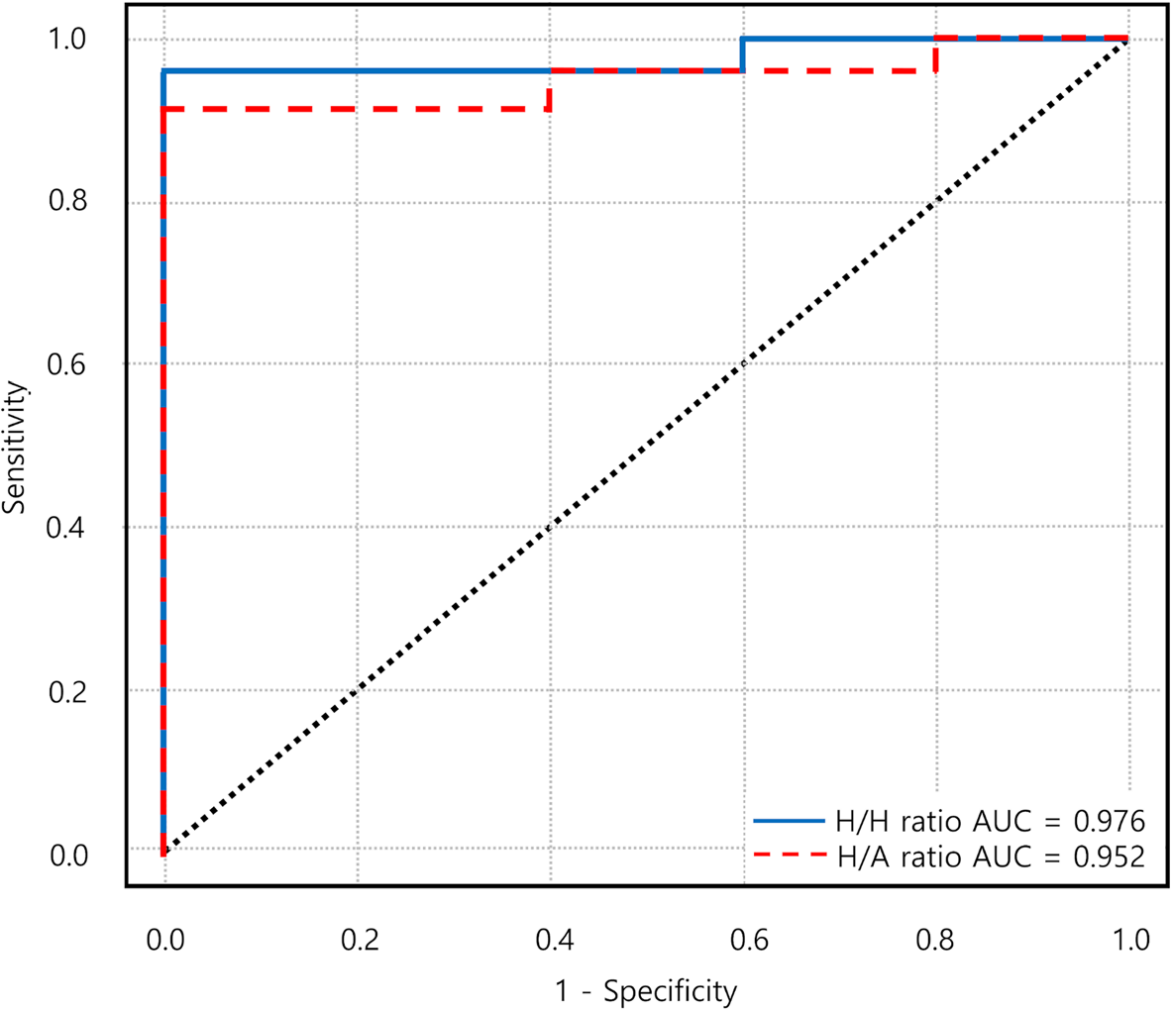
ROC curve plotted from the data of each group. The head-to-head ratio of 0.5 and the head-to-acetabulum ratio of 0.3 is most sensitive for predicting the occurrence of ONFH

At final follow-up, the mean head-to-head uptake ratio of the preoperative SPECT/CT scans was 0.33 (95% CI, 0.28–0.38) in the ONFH group, whereas the mean uptake ratio was 1.30 (95% CI, 1.03–1.57) in the non-ONFH group, showing a statistically significant difference between the two groups (*p* <  0.001). When ROC curve was plotted to define a best cut off value for predicting the ONFH (Fig. 4), a cutoff value of 0.5 was found to have 100% sensitivity and specificity for predicting the ONFH (Table 3).

**Table 3:** Comparison of the head-to-head ratio and head-to-acetabulum ratio between two groups

	ONFH (n = 6)	Non-ONFH (n = 24)	***p***-value
**Uptake of femoral head**			
Affected hip	66.3 (53.7–79.0)	247.6 (134.9–360.3)	0.044
Unaffected hip	201.3 (172.3–230.3)	206.6130.9–282.3)	0.631
**Uptake of acetabulum**			
Affected hip	319.1 (219.6–418.6)	285.7(182.9–388.7)	0.561
Unaffected hip	337.2 (217.2–457.2)	290.2 (194.1–386.4)	0.527
**Head-to-head ratio**	0.33 (0.28–0.38)	1.30 (1.03–1.57)	< 0.001
**Head-to-acetabulum ratio**	0.22 (0.18–0.26)	0.93 (0.72–1.14)	< 0.001

*ONFH* osteonecrosis of the femur head

#### Evaluation of head-to-acetabulum uptake ratio

Six out of 30 patients had limitations in measuring the existing head-to-head ratio. (status post arthroplasty of the contralateral hip; 4 patients, ONFH of the contralateral hip; 1patient, osteoarthritis of the contralateral hip; 1 patient). The mean uptake ratio of six avascular femoral heads to the ipsilateral acetabular dome was 0.22 (95% CI: 0.18–0.26), and the mean uptake ratio of 24 intact femoral heads was 0.93 (95% CI: 0.72–1.14), showing a statistically significant difference (*p* <  0.001). ROC curve was also plotted to define the best cut off value of head-to-acetabulum uptake ratio for predicting the ONFH (Fig. 4). At cut off value of 0.3, the sensitivity and specificity were 85.7 and 100%, respectively (Table 3). When AUC of two ROC curves was compared using DeLong’s algorithm, both predicting models showed no statistical difference (*p* = 0.420) [25].

## Discussion

Scintigraphy has its advantage in the early detection of perfusion or metabolism defects before radiologic abnormalities would appear. Scintigraphy can reflect perfusion defects within 24 h if the test is available and also diagnose and predict ONFH about 14 months earlier than simple radiography [19, 26]. Meyers et al. reported a 95% diagnostic accuracy of scintigraphy in predicting avascular necrosis in his prospective study on FNF patients [27]. Turner et al. also conducted a study with 30 FNF cases using Technetium-99 m scintigraphy and approximated the accuracy of the test at 93% [28].

The diagnostic accuracy for the qualitative evaluation of ONFH using SPECT in our study was 83.3% and was suboptimal compared to other studies [27, 28]. Meyer et al., however, included variable indications besides FNF, such as traumatic hip dislocation or idiopathic ischemic necrosis of the femoral head, in assessing diagnostic accuracy [27]. In the results of Turner et al., there were two patients with absent vascular activity in the bilateral femoral head [28]. Depending on the interpretation of these two patients, the diagnostic accuracy may vary from 93 to 86%. In our study, there was one female patient (13-years-old at diagnosis) whose preoperative perfusion status was viable despite the displacement (Garden type 3 at initial diagnosis). Serial clinical and radiological follow-up results showed favorable outcomes but the patient was later diagnosed with ONFH at a postoperative eight-month follow-up. Considering variable clinical factors related to osteonecrosis, such as the patient’s age, the degree of displacement, the effect of surgical reduction, and the revascularization potential, the preoperative evaluation of perfusion status should be more complicated. Furthermore, there is still no clear guidelines available for displaced FNF patients, and subjective decisions or the surgeon’s preference are used instead. Therefore, a rationale to increase the diagnostic accuracy and positive predictive value of preoperative SPECT/CT in assessing femoral head perfusion status is needed.

Various attempts have been made to predict ONFH by relative quantification. Stromqvist et al. reported that an uptake ratio of the lesion to the unaffected side below 0.9 preoperatively, or 1.0 postoperatively, indicated a high risk of ONFH in displaced FNF patients [29]. Holmberg and Thorngren reported that bone union was expected when the uptake ratio to the unaffected side was over 0.90, whereas complicated outcomes were found under 0.90 [30]. Despite the virtues, conventional planar bone scintigraphy produces poor quality images that cannot provide precise anatomical information, resulting in low specificity. Planar bone scintigraphy also produces a summated 2-dimensional image that can lead to false-negative results by overlying and adjacent bony structures. In this study, therefore, we tried to reveal the predictive value of SPECT/CT based on quantitative analysis of the perfusion status of the femoral head using an ROI.

Quantitative analysis using the head-to-head ratio (with a cutoff value of 0.5) in SPECT/CT showed a high diagnostic accuracy of 100% in predicting osteonecrosis, and the sensitivity and specificity were both 100%. The results were similar to the head-to-acetabulum ratio (with a cutoff value of 0.3), showing a diagnostic accuracy of 96.7%, sensitivity and specificity of 85.7 and 100%, respectively. The concept of ROI in quantitative analysis helps to localize the optimal anatomical area for diagnosis and overcome the distortion of the perfusion status. Using the ipsilateral acetabular dome as a reference point for ROI is useful in certain patients whose perfusion status of the contralateral hip is not comparable due to other medication conditions.

There were several limitations to our study. First, the relatively small number of enrolled patients could diminish the reliability of the clinical application of the results. However, our quantitative evaluation results were satisfactory, showing high diagnostic accuracy, as well as sensitivity and specificity. The follow-up duration was also sufficient enough to detect most of the complications related to osteonecrosis. Second, postoperative follow-up with SPECT/CT was not conducted. Several studies reported the optimal timing of the test at two weeks after surgery, as revascularization, particularly after fracture reduction, is maximized at that time [31–33]. We tried to take SPECT/CT scans two weeks and six months postoperatively, but overcorrection related to implants in the attenuation correction process made the analysis impossible. If further studies identify a solution to the problem of attenuation correction, revascularization and changes in perfusion, with or without reduction, as well as their effects on the occurrence of avascular necrosis, could be revealed. Third, the prediction of ONFH was solely based on the preoperative perfusion state. Various factors affecting osteonecrosis, including patient age, the degree of fracture displacement, and the quality of reduction, should be analyzed. Compared to a study by Turner et al., in which half of the patients were Garden type 3 (50%) fractures, type 1 fractures (56.7%) outnumbered other types of fractures in our study [28]. Since the displacement is minimal, it is hard to predict the perfusion status both quantitatively and qualitatively. Finally, the diagnosis of postoperative ONFH could be underestimated since we only used a simple radiograph to diagnose the occurrence of postoperative ONFH. To overcome this limitation, however, we have radiologically evaluated the patients for a relatively long time with a mean follow-up of 4 years (range, 2.0 to 8.9 years) and also confirmed the diagnosis by bone biopsy of the retrieved femoral head. Since it is widely accepted that ONFH can occur up to 2–3 years after surgery, our longer follow-up would be sufficient to offset this limitation [34].

## Conclusions

Quantitative evaluation of the perfusion status of the femoral head with SPECT/CT improved the diagnostic accuracy in predicting ONFH and could help determine the optimal surgical method and reduce postoperative complications, including reoperation or conversion to arthroplasty. We suggest a cutoff value of 0.5 in the head-to-head ratio and 0.3 in the head-to-acetabular dome ratio as predictive values of post-traumatic ONFH.

## Supplementary information

**Additional file 1.**

## Data Availability

The datasets used and analyzed during the current study are presented as a supplementary file.
